# Performance of Endoscopic Sleeve Gastroplasty by Obesity Class in the United States Clinical Setting

**DOI:** 10.14309/ctg.0000000000000647

**Published:** 2023-10-03

**Authors:** Khushboo Gala, Vitor Brunaldi, Christopher McGowan, Reem Z. Sharaiha, Daniel Maselli, Brandon Vanderwel, Prashant Kedia, Michael Ujiki, Eric Wilson, Eric J. Vargas, Andrew C. Storm, Barham K. Abu Dayyeh

**Affiliations:** 1 Division of Gastroenterology and Hepatology, Mayo Clinic, Rochester, Minnesota, USA;; 2 True You Weight Loss, Cary, North Carolina, USA;; 3 Division of Gastroenterology and Hepatology, Weill Cornell Medicine, New York, New York, USA;; 4 Eviva, Shoreline, Washington, USA;; 5 Methodist Dallas Medical Center, Dallas, Texas, USA;; 6 NorthShore University Health System, Evanston, Illinois, USA;; 7 University of Texas Health Science Center—Houston, Houston, Texas, USA.

**Keywords:** endoscopic sleeve gastroplasty, obesity, severe obesity

## Abstract

**INTRODUCTION:**

Endoscopic sleeve gastroplasty (ESG) has gained popularity over the past decade and has been adopted in both academic and private institutions globally. We present outcomes of the largest cohort of patients from the United States undergoing ESG and evaluate these according to obesity class.

**METHODS:**

We performed a retrospective analysis of adult patients who underwent ESG. Medical information was abstracted from the electronic record with weight records up to 2 years after ESG. Percent total body weight loss (%TBWL) at 6, 12, 18, and 24 months was calculated based on baseline weight at the procedure. SPSS (version 29.0) was used for all statistical analyses.

**RESULTS:**

A total of 1,506 patients from 7 sites were included (501 Class I obesity, 546 Class II, and 459 Class III). Baseline demographics differed according to obesity class due to differences in age, body mass index (BMI), height, sex distribution, and race. As early as 6 months post-ESG, mean BMI for each class dropped to the next lower class and remained there through 2 years. %TWBL achieved in the Class III group was significantly greater when compared with other classes at all time points. At 12 months, 83.2% and 60.9% of patients had ≥10% and ≥15% TBWL for all classes. There were no differences in adverse events between classes.

**DISCUSSION:**

Real-world data from a large cohort of patients of all BMI classes across the United States shows significant and sustained weight loss with ESG. ESG is safe to perform in a higher obesity class with acceptable midterm efficacy.

## INTRODUCTION

Obesity in a non-Asian population is classified based on body mass index (BMI) as Class I (BMI 30–34.9), Class II (BMI 35–39.9), and Class III (BMI 40 and higher, also known as severe obesity). There has been a steady increase in the age-adjusted prevalence of obesity and severe obesity, such that by 2030, 25% and 50% of the US population will be affected by severe obesity and obesity, respectively (1,2). It is known that higher obesity classes, especially Class III, are associated with higher comorbidities and a larger burden on health care (3–5). Treating severe obesity effectively and safely is extremely challenging. Bariatric surgery is the most effective treatment for patients with an advanced BMI class; however, it is used in <1% of the eligible population (6). Barriers to bariatric surgery include health policy, insurance coverage, and other financial issues as well as limited awareness of bariatric surgery, stigma, and missed opportunities for appropriate referrals by primary care providers (7). For patients, barriers to bariatric surgery include fear of surgery and lifestyle change (8). Finally, there is a subset of patients who are too sick to undergo surgery. For many of these patients, endoscopic bariatric therapies provide an effective, minimally invasive, and less anatomy-altering solution.

Endoscopic sleeve gastroplasty (ESG) has gained significant popularity over the past 10 years from its initial development (9). It uses an endoscopic suturing device to imbricate the gastric body and significantly reduce gastric volume. ESG has been shown to be a safe and effective treatment option for obesity and was cleared by the US Food and Drug Administration (FDA) for this indication in July 2022. Overall, ESG has been shown to achieve >15% total body weight loss (TBWL), sustained over 18–24 months, with a low incidence of serious adverse events (10). In the United States, ESG is increasingly being used across a spectrum of bariatric practices, including academic and private practice settings, and by surgeons and gastroenterologists (11,12). This article reports a real-world clinical experience of the largest cohort of patients from the United States that have undergone ESG. We aimed to evaluate weight loss and safety outcomes by obesity class.

## METHODS

### Study design and eligibility criteria

We performed a retrospective analysis of patients who underwent ESG from different sites across the United States from January 2013 through August 2022. These sites included academic and private institutions, and procedures were performed by gastroenterologists and bariatric surgeons. The primary institutional review board approved the study and waived the need for informed consent for data collection owing to its minimal-risk nature. IRB approval was also obtained at other sites. All adult patients who had undergone ESG using a standard technique with full-thickness suturing using the OverStitch system (Apollo Endosurgery, Austin, TX) with the primary goal of weight loss were included. ESG in cases with BMI >50 was performed outside of the indicated FDA label for use in patients with BMI 30–50 kg/m^2^.

### Data collection and study end points

Patient demographic and medical information were abstracted from the electronic medical records. Indications and technical details of each procedure were collected. Baseline weight was defined as weight on the day of the intervention. Weight was recorded at baseline and 6, 12, 18, and 24 months after the procedure. Intraprocedural and postprocedural adverse events were recorded. Percent total body weight loss (%TBWL) and %excess weight loss (%EWL, based on BMI = 25 kg/m^2^) were calculated based on baseline weight at the procedure. Responders to treatment were defined as reaching a predetermined threshold for %TBWL at 12 and 24 months. Each responder group includes the number of patients who satisfy those criteria, that is, 10% is all patients who achieved ≥10% TBWL (i.e., all responders). The patients were divided into groups based on their BMI class (Class I obesity, Class II obesity, and Class III obesity).

The primary end point of this study was to assess weight loss outcomes (BMI and %TBWL) between BMI classes at 6, 12, 18, and 24 months after the procedure. Secondary objectives for this study included evaluation of responders to treatment among BMI classes and evaluation of adverse events according to BMI classes.

### Statistical analysis

All continuous data are summarized as the means and 95% confidence intervals. Analyses by obesity class at each follow-up period were performed using 1-way analysis of variance with Bonferroni correction to identify significant comparisons or χ^2^. Significance was defined as *P* < 0.05. Imputation methods were used to evaluate the impact of missing data from the results. Last observation carried forward reported the responder level at the last recorded visit, and if the subject did not return for any visit (12.1% or 76/626), the subject was not considered a responder. Best-case scenario was defined such that those subjects who had missing data were considered a responder at 10% TBWL while worst-case scenario was defined such that those subjects who had missing data were not considered responders. SPSS (IBM SPSS Statistics for Windows, version 29.0, released 2021; IBM, Armonk, NY) was used for all statistical analyses.

## RESULTS

### Baseline characteristics

At baseline, our cohort comprised 1,506 patients who were predominantly female (84.5%) and White (69.6%). The mean age was 45.68 ± 10.25 years. Mean weight and BMI at baseline were 107.3 ± 21.42 kg and 38.43 ± 6.22, respectively.

The cohort included 501 patients with Class I obesity, 546 patients with Class II obesity, and 459 patients with Class III obesity. Baseline demographics differed by obesity class (Table 1). Although most patients were women, the percentage of men increased with obesity class (*P* < 0.001). There was a higher number of Whites than all other races; however, the proportion of African Americans to Whites increased with rising obesity class (*P* = 0.006). We found no difference between the 2 provider specialties about patient selection by obesity class (*P* = 0.49). Patients with Class III obesity were significantly younger than patients in other obesity classes (*P* < 0.001). Baseline anthropometric characteristics were different for each obesity class, as was expected because of the expected differences in weight and BMI. However, we also identified differences concerning height and ideal weight among groups (*P* < 0.001). Both height and ideal weight (based on the height difference) were higher for those patients with Class III obesity, but not clinically significant (<1 cm of mean difference in height, <1.6 kg of mean difference in ideal weight).

**Table 1.** Baseline demographics by obesity class

Description	Class I (N = 501)	Class II (N = 546)	Class III (N = 459)	Total (N = 1,506)
Sex, n (%)			^a^	^a^
Male	48 (9.6)	75 (13.7)	110 (24.0)	233 (15.5)
Female	453 (90.4)	471 (86.3)	348 (76.0)	1,272 (84.5)
Race, n (%)				
N	431	492	393	1,316
White	323 (74.9)	348 (70.7)	245 (62.3)	916 (69.6)
African American	47 (10.9)	77 (15.7)	90 (22.9)	214 (16.3)
Asian	8 (1.9)	9 (1.8)	6 (1.5)	23 (1.7)
Hispanic	8 (1.9)	10 (2.0)	7 (1.8)	25 (1.9)
Other	10 (2.3)	10 (2.0)	7 (1.8)	27 (2.1)
Not reported	35 (8.1)	38 (2.9)	38 (2.9)	111 (8.4)
Provider type, n (%)				
Bariatric surgeon	85 (17.0)	78 (14.3)	72 (15.7)	235 (15.6)
Gastroenterologist	416 (83.0)	468 (85.7)	387 (84.3)	1,271 (84.4)
Age (yr)			^a^	^a^
Mean ± SD	47.01 ± 10.03	45.77 ± 10.20	44.11 ± 10.35	45.68 ± 10.25
Min, max	19.0, 72.1	17.5, 73.6	17.2, 72.7	17.2, 73.6
95% CI	46.1, 47.9	44.9, 46.6	43.2, 45.1	45.2, 46.2
Height (m)				
Mean ± SD	1.66 ± 0.08	1.67 ± 0.08	1.68 ± 0.10	1.67 ± 0.09
Min, max	1.5, 2.0	1.4, 1.9	1.3, 2.0	1.3, 2.0
95% CI	1.65, 1.67	1.66, 1.67	1.67, 1.69	1.66, 1.67
Weight (kg)				
Mean ± SD	90.7 ± 9.02	104.0 ± 11.33	129.4 ± 21.54	107.3 ± 21.42
Min, max	66.2, 129.0	76.2, 137.4	89.4, 240.4	66.2, 240.4
95% CI	89.9, 91.5	103.0, 104.9	127.4, 131.4	106.2, 108.4
Ideal weight (kg)				
Mean ± SD	69.0 ± 6.38	69.5 ± 6.98	70.9 ± 8.62	69.7 ± 7.37
Min, max	53.3, 95.6	50.6, 90.7	42.0, 98.1	42.9, 8.1
95% CI	68.5, 69.6	68.9, 70.0	70.1, 71.7	69.4, 70.1
Excess weight (kg)				
Mean ± SD	21.7 ± 4.13	34.5 ± 5.45	58.5 ± 16.52	37.6 ± 17.90
Min, max	12.0, 35.83	24.1, 51.5	33.9, 152.1	12.0, 152.1
95% CI	21.4, 22.1	34.0, 35.0	57.0, 60.0	36.7, 38.5
BMI (kg/m^2^)				
Mean ± SD	32.87 ± 1.34	37.41 ± 1.47	45.7039 ± 5.88	38.43 ± 6.22
Min, max	30.00, 34.97	35.00, 39.99	40.01, 111.10	30.00, 111.10
95% CI	32.75, 32.99	37.29, 37.53	45.16, 46.24	38.11, 38.74

BMI, body mass index; CI, confidence interval.

a One subject has no reported response.

### Weight loss outcomes

**Figure 1.**
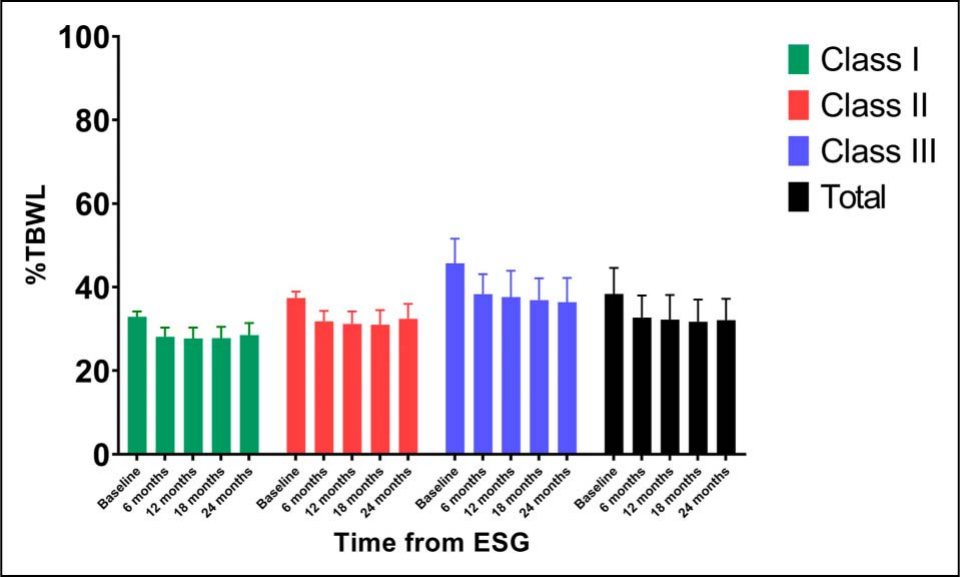
BMI by obesity class and time from the procedure. BMI, body mass index; ESG, endoscopic sleeve gastroplasty; %TBWL, percent total body weight loss.

**Figure 2.**
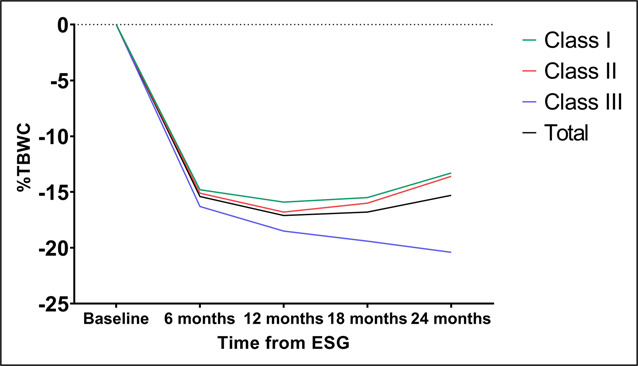
%Total body weight change by obesity class and time from the procedure. ESG, endoscopic sleeve gastroplasty; %TBWL, percent total body weight loss.

Weight loss outcomes are described in Figures 1 and 2. The mean %TBWL in the cohort was 15.4 (15.0, 15.7), 17.1 (16.6, 17.6), 16.8 (16.0, 17.6), and 15.3 (14.3, 16.5) for 6, 12, 18, and 24 months, respectively. A total of 83.2% and 69.9% of patients achieved >10% TBWL at 12 and 24 months, respectively.

### Weight loss outcomes by obesity class

Weight loss parameters by obesity class are reported for BMI and %TBWL (Figures 1 and 2, respectively). The mean baseline BMI was 32.87 ± 1.3, 37.41 ± 1.5, and 45.70 ± 5.9 for Class I, II, and III, respectively. At 6 months, the mean BMI and 95% confidence interval for each class dropped to the next lower class and remained there throughout the 2-year follow-up period. This remained true while analyzing BMI data for subjects with complete visit data (see Supplementary Table 1, Supplementary Digital Content 1, http://links.lww.com/CTG/B26).

There was a significant difference in %TBWL about obesity class (*P* = 0.001 for all time points), and the mean %TBWL in the Class III group was significantly greater compared with all other classes at all time points. Interestingly, %TBWL continually improved throughout follow-up for patients with Class III obesity at baseline, such that by 24 months the mean %TBWL was >20%. As with BMI, these observations held true when data from subjects with complete visit data were analyzed. Weight loss for patients with Class III obesity plateaued at around 20% TBWL at 12 months and beyond (see Supplementary Table 2, Supplementary Digital Content 1, http://links.lww.com/CTG/B26).

Responders to treatment are described in Table 2. At 12 months, >80% of patients of all obesity classes achieved >10% TBWL. More than a third of the total cohort achieved >20% TBWL. Patients with Class III obesity had the highest response rate at all levels.

**Table 2.** Responders at 12-month follow-up by obesity class

Responder definition	Class I (N = 304)	Class II (N = 336)	Class III (N = 311)	Total (N = 951)
10%	80.3% (244)	82.4% (277)	86.8% (270)	83.2% (791)
15%	54.3% (165)	61.9% (208)	66.2% (206)	60.9% (579)
20%	24.0% (73)	33.0% (111)	37.0% (115)	31.4% (299)
25%	10.2% (31)	11.3% (38)	19.0% (59)	13.5% (128)
30%	4.3% (13)	4.2% (14)	8.0% (25)	5.5% (52)
40%	0.3% (1)	0.6% (2)	2.6% (8)	1.2% (11)

At 24 months, 58.4% (880/1,506) had not yet reached the expected visit window (24 ± 3 months). Follow-up rate for those subjects who were expected to complete a 24-month visit was 54.2% (339/626). The responders at 24 months for those subjects who completed the visit is presented in Supplementary Table 3A (see Supplementary Digital Content 1, http://links.lww.com/CTG/B26). Completer's analysis shows that >60% of patients in all groups were able to achieve 10% TBWL. Of note, 86.8% patients with Class III obesity were able to achieve that response. This difference is also noted in the group of patients who achieved a 20% TBWL, where patients with Class III obesity have a 44% response rate compared with Classes I and II (19.8% and 23.6%, respectively).

Imputation analyses were performed to handle missing data. Only subjects who should have completed a 24-month visit were included, which included last observation carried forward (see Supplementary Table 3B, Supplementary Digital Content 1, http://links.lww.com/CTG/B26), best-case scenario (see Supplementary Table 3C, Supplementary Digital Content 1, http://links.lww.com/CTG/B26), and worst-case scenario (see Supplementary Table 3D, Supplementary Digital Content 1, http://links.lww.com/CTG/B26). Responders are slightly lower with last observation carried forward analysis compared with completer's analysis. The best-case and worst-case scenario report that overall, 83.9% or 37.9% patients, respectively, are able to achieve >10% TBWL.

### Adverse events

Adverse events are described in Table 3. Only events that were reported as either device or procedure related are included in the analysis below. Two hundred fifty-six patients experienced 360 events, most of which occurred during the first 30 days after the procedure (337/360). Among those, 312 events were reported within 7 days of the procedure. Thirty-nine patients (2.6%) experienced at least 1 adverse event that required hospitalization (51 events total) for treatment. Most of those events (44/51) were addressed with medications for symptom management and fluid replacement. Three of the 51 events required surgical interventions.

**Table 3.** Overview of adverse events by obesity class: patient rate

Adverse event	Class I (N = 501)	Class II (N = 546)	Class III (N = 459)	Total (N = 1,506)
Bleeding from suture site	1 (0.2%)	0	0	1 (0.1%)
Chest pain	0	2 (0.4%)	0	2 (0.1%)
Constipation	2 (0.4%)	2 (0.4%)	3 (0.7%)	7 (0.5%)
Dehydration	4 (0.8%)	4 (0.7%)	3 (0.7%)	11 (0.7%)
Dizziness	1 (0.2%)	1 (0.2%)	0	2 (0.1%)
Eructation	0	1 (0.2%)	0	1 (0.1%)
Gastric leak	0	0	1 (0.2%)	1 (0.1%)
GERD	4 (0.8%)	4 (0.7%)	1 (0.2%)	9 (0.6%)
Headache	0	2 (0.4%)	0	2 (0.1%)
Hematemesis	2 (0.4%)	3 (0.5%)	0	5 (0.3%)
Nausea/vomiting	62 (12.4%)	62 (11.4%)	58 (12.6%)	182 (12.1%)
Oral thrush	0	1 (0.2%)	0	1 (0.1%)
Pain	48 (9.6%)	33 (6.0%)	35 (7.6%)	116 (7.7%)
Perforation	2 (0.4%)	2 (0.4%)	0	4 (0.3%)
Perigastric abscess	1 (0.2%)	1 (0.2%)	0	2 (0.1%)
Shortness of breath	1 (0.2%)	0	0	1 (0.1%)
Somnolence/med effect	0	1 (0.2%)	0	1 (0.1%)

GERD, gastroesophageal reflux disease.

When evaluated by obesity class, there were no differences in incidence of adverse events. It was observed that

- class I: 98 patients (19.6%) experienced 134 events,
- class II: 85 patients (15.6%) experienced 125 events, and
- class III: 73 patients (15.9%) experienced 101 events.

## DISCUSSION

Our data, which comprise the largest cohort of patients from the United States who have undergone ESG, show significant and sustained weight loss in the midterm. A mean %TBWL of 15.4% is achieved at 6 months, sustained at 12 and 24 months. The %TBWL at 12 months is 17.1%, which is higher than the 13.6% that was seen in the Multicenter ESG Randomized Interventional Trial (MERIT), the first randomized controlled trial proving the efficacy of ESG (13). It is comparable to data from the largest published meta-analysis of ESG that showed a mean %TBWL of 15.1%, 16.5%, and 17.2% at 6, 12, and 18–24 months, respectively (10). These differences between trial and real-world data could be attributable to multiple factors. First, most patients pay out-of-pocket outside of clinical trials, which may influence their motivation, attrition, and weight loss outcomes. A significant placebo and nocebo effect has been seen in trials relating to obesity, which may explain these differences (14). Finally, adjunctive pharmacotherapy was used in a minority of patients which may influence outcomes. Outcomes from our cohort are more representative of what we can expect from ESG when used in a widespread, commercial setting.

This higher %TBWL seen in our cohort seems to be driven by patients with Class III obesity. At every time point, these patients had a significantly higher %TBWL compared with other BMI classes. At 24 months, these patients had a significantly higher mean %TBWL of 20.4%, compared with those with Class I obesity (13.3%) and Class II obesity (13.6%). Interestingly, patients with Class III obesity had a continued trajectory of weight loss, compared with other groups. Notably, patients of all BMI classes were able to drop to the next BMI class. However, ongoing improvement in BMI until the end of the follow-up period was seen only in patients with Class III obesity. Of note, patients with Class III obesity were significantly younger and taller than their counterparts in Class I and II obesity. This may signal a more aggressive form of disease, which has often been described as Class III obesity is distinctly different from other classes in adiposity, metabolic burden, and complexity of care required, and wherein patients may require therapy at an earlier stage (15). These differences in outcomes to the same procedure add to the remarkable heterogeneity observed among individuals in different obesity classes. It is encouraging to see that ESG is effective in this situation.

The safety data in all obesity classes in our cohort were reassuring. In general, only 2.6% of patients had an adverse event requiring hospitalization. Most of these events (86%) were for symptom management and/or fluid replacement; only 3 events required a surgical intervention. Results from our cohort are comparable to safety data from large meta-analyses, which quote severe adverse event rates of 2%–2.5% (16). This represents an excellent safety profile for this procedure, without any evidence of increased adverse events in patients with Class III obesity. The safety profile of ESG in severe obesity has been described previously only in small case series. There is a report of 5 patients with super obesity in Germany, with BMIs ranging from 51 to 72 kg/m^2^ and with contraindications to surgery, which focuses primarily on the safety and feasibility of the procedure and only reports weight loss of 3 months. Finally, a case series from Brazil describes excellent outcomes in 24 patients with high risk, patients with BMI >50, and patients contraindicated to abdominal surgeries showed reasonable outcomes (%TBWL of 12.2% ± 8.9% and 1 severe adverse event (17)).

ESG in severe obesity has been studied with a shorter follow-up duration in a European cohort. Lopez-Nava et al presented 1-year weight loss data on 396 patients in Spain (18). A total of 146 patients had Class III obesity, with a slightly lower mean BMI than our cohort (44.5 ± 3.8 vs 45.7 ± 5.9). Patients with Class III obesity in the Spanish cohort lost 20.5% TBWL at 1 year with higher weight loss in Class III obesity, which is slightly higher than the 18.4% TBWL seen in patients with severe obesity in our cohort. This difference may be attributable to the larger size of our cohort (459 vs 146 patients with severe obesity) and differences in weight loss outcomes between populations. However, in a cohort of 233 patients from Brazil, Neto et al show conflicting results of lower %TBWL seen in patients with higher BMI (19). It is seen here that patients with Class II obesity have a %TBWL of 19.3%, compared with 20.1% seen in Class I obesity. Class III obesity patients were not studied in the Brazilian cohort. Bariatric surgical literature suggests that patients with higher preoperative BMI may have lesser weight loss with bariatric surgery; however, this is often reported for excess weight loss and may not be generalizable (20,21).

Our cohort shows a robust %TBWL of about 18%–20% in severe obesity; this is lower than that obtained by bariatric surgery (17). Even with bariatric surgery available, there certainly is a position for ESG in the management of severe obesity. First, being minimally invasive and anatomy preserving, ESG may represent an ideal solution for patients and providers who are reluctant concerning bariatric surgery. In addition, ESG has been shown to be an exceedingly safe procedure, with a superior safety profile compared with laparoscopic sleeve gastrectomy (16,22). With the available data, this safety profile is mirrored even in patients with severe obesity, although it is well known that there is an increased risk of perioperative complications with surgical interventions in this patient population. Hence, ESG may represent a lower risk, reproducible option for this patient population. Furthermore, repeating either endoscopic or bariatric surgery remains an option in patients after ESG. There are limited data that show that weight loss after ESG may be enhanced with pharmacotherapy, even reaching almost 25% TBWL in 1 study; however, most studies show that adjunct pharmacotherapy generally only serves to reach target weight loss rather than additional weight loss (23). Future research into enhancing weight loss outcomes after ESG is warranted.

Our study represents the first report of a large cohort of patients from the United States with severe obesity managed with ESG. Our data are gathered from 7 different sites, both academic and private, with ESGs performed by both gastroenterologists and bariatric surgeons. As such, it is fairly representative of the current practice of ESG in the United States. It is also the largest cohort of patients who have undergone this procedure in the United States. Even globally, there are extremely limited data with this size of cohort (Neto et al (24) in Brazil, 1,828 patients with 1-year data). We have follow-up data for 2 years, which is indicative of midterm efficacy.

Our study has several limitations. Because of the retrospective nature, the multicentric nature of our cohort, and relative novelty of the procedure, we were unable to obtain outcomes for greater than 2 years. We also have loss of follow-up data (339 patients analyzed of 1,506 at 2 years), which is attributable to real-world practices by patients and providers. One must consider that this may be due to achievement of the weight loss goals, although there is ample clinical evidence that supports continued follow-up as a strong predictor of good outcomes. We have performed completers and imputation analyses to compensate for this. Again, because of the retrospective nature of this study, safety data have not been gathered in a standardized fashion but reported per each institution. The effect of additional therapies, such as intensive lifestyle modification or antiobesity medication, was not considered during our analysis. However, we believe that these limitations will not affect the reliability of our overall results because it has been noted in several studies that postprocedural pharmacotherapy in patients after ESG is able to prevent further weight gain, but did not lead to significant additional weight loss (25,26).

Traditionally, ESG has been proposed as a treatment choice for patients with Class I and II obesity because of its modest weight loss outcomes. However, our data show a %TBWL crossing 20% in patients with Class III disease, which may push the envelope of perceived utility of ESG. Studies examining longer term efficacy are key to understanding the role of ESG in patients with severe obesity.

## CONFLICTS OF INTEREST

**Guarantor of the article:** Barham K. Abu Dayyeh, MD, MPH.

**Specific author contributions:** K.G., V.B. and B.K.A.D.: conceptualized and designed the study. C.M., R.Z.S., D.M., B.V., P.K., M.U., E.W., E.J.V., A.C.S., and B.K.A.D.: contributed to patient accrual, procedures, and data collection. K.G. and V.B.: performed data analysis. K.G., V.B., A.C.S., and B.K.A.D.: wrote and reviewed the manuscript. All authors reviewed the final draft before submission.

**Financial support:** None to report.

**Potential competing interests:** A.C.S. has research grants from Apollo Endosurgery, Boston Scientific, Endogenex, EnteraSense, and OnePass, and is a consultant for Apollo Endosurgery, Boston Scientific, Endogenex, Endo-TAGSS, MGI Medical, Olympus, Intuitive, Medtronic, and Microtech. M.U. is a board member for Boston Scientific, is a paid consultant for Olympus and Cook, and receives payment for lectures from Medtronic, Gore, and Erbe. P.K. is a consultant for Boston Scientific, Medtronic, and Olympus. R.Z.S. is a consultant for Boston Scientific, Cook Medical, and Lumendi. B.V. is a consultant for Apollo Endosurgery. D.M. is a consultant for Apollo Endosurgery. B.K.A.D. is a consultant for DyaMx, Boston Scientific, USGI Medical, and Endo-TAGSS; gets research support from Boston Scientific, USGI Medical, Apollo Endosurgery, Spatz Medical, GI Dynamics, Cairn Diagnostics, Aspire Bariatrics, and Medtronic; and is a speaker for Johnson and Johnson, Endogastric Solutions, and Olympus. Other authors do not have a conflict of interest or disclosures.

**Study Highlights**

**WHAT IS KNOWN**

✓ Patients with severe obesity have therapeutic options generally limited to bariatric surgery.
✓ Endoscopic sleeve gastroplasty (ESG) is safe and effective therapy for obesity.

**WHAT IS NEW HERE**

✓ Weight loss after ESG is more pronounced in patients with Class III obesity and reaches >20% at 2 years.
✓ ESG is equally safe in patients with all obesity classes.

## Supplementary Material
