# Supplementary material for: Performance of Endoscopic Sleeve Gastroplasty by Obesity Class in the United States Clinical Setting
Source: Clin Transl Gastroenterol. 2023 Oct 3;15(1):e00647. doi: 10.14309/ctg.0000000000000647 (PMC10810569; doi:10.14309/ctg.0000000000000647)
Supplement: Supplementary file 1 [file ct9-15-e00647-s001.docx]

Supplementary Table 1: BMI by Obesity Class and Time from Procedure (subjects with complete visit data only)

| **Time from Procedure** | **Class I**  **(N=93)** | **Class II**  **(N=111)** | **Class III**  **(N=76)** | **Total**  **(N=280)** |
| --- | --- | --- | --- | --- |
| **Baseline** |  |  |  |  |
| Mean (SD) | 32.76 (1.27) | 37.57 (1.46) | 45.82 (4.75) | 38.21 (5.77) |
| Min, Max | 30.10, 34.97 | 35.00, 39.99 | 40.03, 68.05 | 30.10, 68.05 |
| 95% CI | 32.50, 33.01 | 37.28, 37.84 | 44.73, 46.91 | 37.53, 38.89 |
| **6 months** |  |  |  |  |
| Mean (SD) | 27.65 (2.12) | 31.57 (2.66) | 37.60 (4.22) | 31.90 (4.89) |
| Min, Max | 22.59, 34.37 | 24.80, 40.14 | 30.07, 51.36 | 22.59, 51.36 |
| 95% CI | 27.21, 28.08 | 31.07, 32.07 | 36.63, 38.56 | 31.33, 32.48 |
| **12 months** |  |  |  |  |
| Mean (SD) | 27.50 (2.51) | 31.32 (3.16) | 35.89 (5.44) | 31.29 (4.95) |
| Min, Max | 19.79, 33.48 | 22.60, 41.18 | 21.52, 55.34 | 19.79, 55.35 |
| 95% CI | 26.98, 28.01 | 30.73, 31.31 | 36.65, 37.14 | 31.88, 31.05 |
| **18 months** |  |  |  |  |
| Mean (SD) | 27.83 (2.55) | 31.81 (3.46) | 36.33 (5.231804) | 31.71 (5.01) |
| Min, Max | 19.20, 34.72 | 22.18, 41.52 | 24.56, 56.11 | 19.20, 56.11 |
| 95% CI | 27.30, 28.36 | 31.16, 32.46 | 35.13, 37.52 | 31.13. 32.30 |
| **24 months** |  |  |  |  |
| Mean (SD) | 28.30 (2.62) | 32.05 (3.54) | 36.11 (5.68) | 31.91 (5.00) |
| Min, Max | 19.64, 34.72 | 22.71, 42.93 | 23.62, 56.24 | 19.64, 56.24 |
| 95% CI | 27.76, 28.84 | 31.38, 32.72 | 34.81, 37.41 | 31.32, 32.50 |

Supplementary Table 2: %TBWL by Obesity Class and Time from Procedure (subject with complete visit data only)

| **Time from Procedure** | **Class I**  **(N=93)** | **Class II**  **(N=111)** | **Class III**  **(N=76)** | **Total**  **(N=280)** |
| --- | --- | --- | --- | --- |
| **6 months** |  |  |  |  |
| Mean (SD) | 15.6 (5.93) | 16.0 (6.13) | 17.7 (7.15) | 16.3 (6.40) |
| Min, Max | -4.9, 28.8 | -0.9, 34.5 | -2.5, 44.3 | -4.9, 44.3 |
| 95% CI | 14.4, 16.8 | 14.8, 17.1 | 16.1, 19.4 | 15.6, 17.1 |
| **12 months** |  |  |  |  |
| Mean (SD) | 16.0 (7.18) | 16.6 (8.04) | 21.5 (10.34) | 17.7 (8.74) |
| Min, Max | 0.5, 35.3 | -3.5, 41.5 | -3.9, 49.7 | -3.9, 48.7 |
| 95% CI | 14.6, 17.5 | 15.1, 18.1 | 19.1, 23.8 | 16.7, 18.8 |
| **18 months** |  |  |  |  |
| Mean (SD) | 15.0 (7.23) | 15.3 (8.94) | 20.5 (10.29) | 16.6 (9.10) |
| Min, Max | -0.5, 37.2 | -4.6, 42.6 | -0.6, 48.0 | -4.6, 48.0 |
| 95% CI | 13.5, 16.5 | 13.6, 16.9 | 18.1, 22.8 | 15.5, 17.7 |
| **24 months** |  |  |  |  |
| Mean (SD) | 13.6 (7.54) | 14.6 (9.35) | 21.0 (10.90) | 16.0 (9.73) |
| Min, Max | -4.9, 35.8 | -11.3, 41.2 | -2.5, 50.0 | -11.3, 50.0 |
| 95% CI | 12.0, 15.1 | 12.9, 16.4 | 18.5, 23.5 | 14.9, 17.2 |

Supplementary Table 3. Responders at 24-month follow-up by Obesity Class

| **Responder Definition** | **Class I**  **(N=121)** | **Class II**  **(N=127)** | **Class III**  **(N=91)** | **Total**  **(N=339)** |
| --- | --- | --- | --- | --- |
| **A: Completers** | | | | |
| 10% | 62.8% (76) | 64.6% (82) | 86.8% (79) | 69.9% (237) |
| 15% | 38.0% (46) | 40.9% (52) | 64.8% (59) | 46.3% (157) |
| 20% | 19.8% (24) | 23.6% (30) | 44.0% (40) | 27.7% (94) |
| 25% | 7.4% (9) | 11.8% (15) | 33.0% (30) | 15.9% (54) |
| 30% | 3.3% (4) | 3.9% (5) | 22.0% (20) | 8.6% (29) |
| 40% | 0.8% (1) | 2.4% (3) | 6.6% (6) | 2.9% (10) |
| **B: Last Observation Carried Forward** | | | | |
|  | **Class I**  **(N=216)** | **Class II**  **(N=230)** | **Class III**  **(N=180)** | **Total**  **(N=626)** |
| 10% | 56.0% (121) | 58.7% (135) | 72.8% (131) | 61.8% (387) |
| 15% | 32.9% (71) | 36.5% (84) | 52.2% (94) | 39.8% (249) |
| 20% | 18.1% (39) | 19.6% (45) | 26.7% (48) | 21.1% (132) |
| 25% | 6.0% (13) | 7.8% (18) | 19.4% (35) | 10.5% (66) |
| 30% | 2.8% (6) | 2.6% (6) | 12.8% (23) | 5.6% (35) |
| 40% | 0.5% (1) | 1.3% (3) | 3.9% (7) | 1.8% (11) |
| **C: Best Case Scenario** | | | | |
|  | **Class I**  **(N=216)** | **Class II**  **(N=230)** | **Class III**  **(N=180)** | **Total**  **(N=626)** |
| 10% | 79.1% (171) | 81.4% (185) | 93.3% (168) | 83.9% (524) |
| 15% | 38.0% (46) | 409% (52) | 64.8% (59) | 46.3% (157) |
| 20% | 19.8% (24) | 23.6% (30) | 44.0% (40) | 27.7% (94) |
| 25% | 7.4% (9) | 11.8% (15) | 33.0% (30) | 15.9% (54) |
| 30% | 3.3% (4) | 3.9% (5) | 22.0% (20) | 8.6% (29) |
| 40% | 0.8% (1) | 2.4% (3) | 6.6% (6) | 2.9% (10) |
| **D: Worst Case Scenario** | | | | |
|  | **Class I**  **(N=216)** | **Class II**  **(N=230)** | **Class III**  **(N=180)** | **Total**  **(N=626)** |
| 10% | 35.2% (76) | 35.6% (82) | 43.9% (79) | 37.9% (237) |
| 15% | 21.3% (46) | 22.6% (52) | 32.8% (59) | 25.1% (157) |
| 20% | 11.1% (24) | 13.0% (30) | 22.2% (40) | 15.0% (94) |
| 25% | 4.2% (9) | 6.5% (15) | 16.7% (30) | 8.6% (54) |
| 30% | 1.9% (4) | 2.2% (5) | 11.1% (20) | 4.6% (29) |
| 40% | 0.5% (1) | 1.3% (3) | 3.3% (6) | 1.6% (10) |


Supplementary methods: Total number of participants by site

1: 249; 2: 216; 3: 164; 4: 47; 5: 35; 6: 784; 7: 11
